# Large Deviations for Nonlocal Stochastic Neural Fields

**DOI:** 10.1186/2190-8567-4-1

**Published:** 2014-04-17

**Authors:** Christian Kuehn, Martin G Riedler

**Affiliations:** 1 Institute for Analysis and Scientific Computing, Vienna University of Technology, 1040, Vienna, Austria; 2 Institute for Stochastics, Johannes Kepler Universität, Altenbergerstraße 69, 4040, Linz, Austria

**Keywords:** Stochastic neural field equations, Nonlocal equations, Large deviation principle, Galerkin approximation

## Abstract

We study the effect of additive noise on integro-differential neural field equations. In particular, we analyze an Amari-type model driven by a *Q*-Wiener process, and focus on noise-induced transitions and escape. We argue that proving a sharp Kramers’ law for neural fields poses substantial difficulties, but that one may transfer techniques from stochastic partial differential equations to establish a large deviation principle (LDP). Then we demonstrate that an efficient finite-dimensional approximation of the stochastic neural field equation can be achieved using a Galerkin method and that the resulting finite-dimensional rate function for the LDP can have a multiscale structure in certain cases. These results form the starting point for an efficient practical computation of the LDP. Our approach also provides the technical basis for further rigorous study of noise-induced transitions in neural fields based on Galerkin approximations.

**Mathematics Subject Classification (2000):** 60F10, 60H15, 65M60, 92C20.

## 1 Introduction

Starting from the classical works of Wilson/Cowan [64] and Amari [1], there has been considerable interest in the analysis of spatiotemporal dynamics of mesoscale models of neural activity. Continuum models for neural fields often take the form of nonlinear integro-differential equations where the integral term can be viewed as a nonlocal interaction term; see [37] for a derivation of neural field models. Stationary states, traveling waves, and pattern formation for neural fields have been studied extensively; see, e.g., [20,29] or the recent review by Bressloff [14] and references therein. 

In this paper, we are going to study a stochastic neural field model. There are several motivations for our approach. In general, it is well known that intra and interneuron [27] dynamics are subject to fluctuations. Many meso or macroscale continuum models have stochastic perturbations due to finite-size effects [38,61]. Therefore, there is certainly a genuine need to develop new techniques to analyze random neural systems [50]. For stochastic neural fields, there is also the direct motivation to understand the relation between noise and short-term working memory [52] as well as noise-induced phenomena [54] in perceptual bistability [62]. Although an eventual goal is to match results from stochastic neural fields to actual cortex data [35], we shall not attempt such a comparison here. However, the techniques we develop could have the potential to make it easier to understand the relation between models and experiments; see Sect. 10 for a more detailed discussion.

There is a relatively small amount of fairly recent work on stochastic neural fields, which we briefly review here. Brackley and Turner [11] study a neural field with a gain function, which has a random firing threshold. Fluctuating gain functions are also considered by Coombes et al. [22]. Bressloff and Webber [15] analyze a stochastic neural field equation with multiplicative noise while Bressloff and Wilkinson [16] study the influence of extrinsic noise on neural fields. In all these works, the focus is on the statistics of traveling waves such as front diffusion and the effects of noise on the wave speed. Hutt et al. [41] study the influence of external fluctuations on Turing bifurcation in neural fields. Kilpatrick and Ermentrout [43] are interested in stationary bump solutions. They observe numerically a noise-induced passage to extinction as well as noise-induced switching of bump solutions and conjecture that “a Kramers’ escape rate calculation” [[43], p. 16] could be applied to stochastic neural fields, but they do not carry out this calculation. In particular, the question is whether one can give a precise estimate of the mean transition time between metastable states for stochastic neural field equations; for a precise statement of the classical Kramers’ law; see Sect. 5, Eq. (32). However, to the best of our knowledge, there seems to be no general Kramers’ law or large deviation principle (LDP) calculation available for continuum neural field models although large deviations have been of recent interest in neuroscience applications [13,33]. It is one of the main goals of this paper to provide the basic steps toward a general theory. 

Although Kramers’ law [5] and LDPs [26,34] are well understood for finite-dimensional stochastic differential equations (SDEs), the work for infinite-dimensional evolution equations is much more recent. In particular, it has been shown very recently that one may extend Kramers’ law to certain stochastic partial differential equations (SPDEs) [4,6,7] driven by space-time white noise. The work of Berglund and Gentz [7] provides a quite general strategy how to “lift” a finite-dimensional Kramers’ law to the SPDE setting using a Galerkin approximation due to Blömker and Jentzen [8]. Since the transfer of PDE techniques to neural fields has been very successful, either directly [51] or indirectly [14,21], one may conjecture that the same strategy also works for SPDEs and stochastic neural fields. 

In this paper, we consider a rate-based (or Amari) neural field model driven by a *Q*-Wiener process *W*

(1) $$dU_{t}(x)=\left[-\alpha U_{t}(x)+\int_{B}w(x,y)f\left(U_{t}(y)\right)dy\right]dt+\epsilon dW_{t}(x)$$

for a trace-class operator *Q*, nonlinear gain function *f*, and an interaction kernel *w*; the technical details and definitions are provided in Sect. 2. Observe that (1) is a relatively general formulation of a nonlocal neural field. Hence, we expect that the techniques developed in this paper carry over to much wider classes of neural fields beyond (1) such as activity-based models.

*Remark 1.1* To avoid confusion, we alert readers familiar with neural fields that the nonlinear gain function *f* in (1) is sometimes also called a “rate function.” However, we reserve “rate function” for a functional, to be denoted later by *I*, arising in the context of an LDP as this convention is standard in the context of LDPs.

Our main goal in the study of (1) is to provide estimates on the mean first passage times between metastable states. In particular, we develop the basic analytical tools to approximate equation (1) as well as its rate function using a finite-dimensional Galerkin approximation. By making the rate function as explicit as possible, we do not only provide a starting point for further analytical work, but also provide a framework for efficient numerical methods to analyze metastable states.

The paper is structured as follows: The motivation for (1) is given in Sect. 3 where a formal calculation shows that a space-time white noise perturbation of the gain function in a deterministic neural field leads to (1). In Sect. 4, we briefly describe important features of the deterministic dynamics for (1) where ϵ=0. In particular, we collect several examples from the literature where the classical Kramers’ stability configuration of bistable stationary states separated by an unstable state occurs for Amari-type neural fields. In Sect. 5, we introduce the notation for Kramers’ law and LDPs and state the main theorem on finite-dimensional rate functions. In Sect. 6, we argue that a direct approach to Kramers’ law via “lifting” for (1) is likely to fail. Although the Amari model has a hidden energy-type structure, we have not been able to generalize the gradient-structure approach for SPDEs to the stochastic Amari model. This raises doubt whether a Kramers’ escape rate calculation can actually be carried out, i.e., whether one may express the prefactor of the mean first-passage in the bistable case explicitly. Based on these considerations, we restrict ourselves to just derive an LDP. In Sect. 7, the LDP is established by a direct transfer of a result known for SPDEs. The disadvantage of this approach is that the resulting rate function is difficult to calculate, analytically or numerically, in practice. Therefore, we establish in Sect. 8 the convergence of a suitable Galerkin approximation for (1). Using this approximation, one may apply results about the LDP for SDEs, which we carry out in Sect. 9. In this context, we also notice that the trace-class noise can induce a multiscale structure of the rate function in certain cases. The last two observations lead to a tractable finite-dimensional approximation of the LDP and hence also an associated finite-dimensional approximation for first-exit time problems. We conclude the paper in Sect. 10 with implications of our work and remarks about future problems.

## 2 Amari-Type Models

In this study, we consider stochastic neural field models with additive noise of the form

(2) $$dU_{t}(x)=\left[-\alpha U_{t}(x)+\int_{B}w(x,y)f\left(U_{t}(y)\right)dy\right]dt+\epsilon dW_{t}(x)$$

for $x\in B\subseteq R^{d}$, a small parameter ϵ>0, and t≥0, where ℬ is bounded and closed. In (2) the solution *U* models the averaged electrical potential generated by neurons at location *x* in an area of the brain ℬ. Neural field equations of the form (2) are called Amari-type equations or a rate-based neural field models. The equation is driven by an adapted space-time stochastic process $W_{t}(x)$ on a filtered probability space $\left(\Omega ,F,{\left(F_{t}\right)}_{t\geq 0},P\right)$. The precise definition of the process *W* will be given below.

The parameter α>0 is the decay rate for the potential, w:B×B→R is a kernel that models the connectivity of neurons at location *x* to neurons at location *y*. Positive values of *w* model excitatory connections and negative values model inhibitory connections. The gain function $f:R\to R_{+}$ relates the potential of neurons to inputs into other neurons. Typically, the gain functions are chosen sigmoidal, for example, (up to affine transformations of the argument) $f(u)={\left(1+e^{-u}\right)}^{-1}$ or f(u)=(tanh(u)+1)/2. These examples of gain functions are bounded, infinitely often differentiable with bounded derivatives. However, throughout the paper, we only make the standing assumption that 

(H1) the gain function *f* is globally Lipschitz continuous on ℝ.

 We may transfer Eq. (2) into the Hilbert space setting of infinite-dimensional stochastic evolution equations [23,56] for the Hilbert space $L^{2}(B)$. Subsequently, brackets 〈⋅,⋅〉 always denote the inner product on this Hilbert space. Moreover, we introduce the following notation. Firstly, *F* denotes the nonlinear Nemytzkii-operator defined from *f*, i.e., F(g)(x)=f(g(x)) for any function $g\in L^{2}(B)$. The condition (H1) implies that $F:L^{2}(B)\to L^{2}(B)$ is a Lipschitz continuous operator. Often, spatially continuous solutions to (2) are also of interest, and thus we note that the Nemytzkii-operator also preserves its Lipschitz continuity on the Banach space C(B) with its norm ${∥g∥}_{0}=\sup_{x\in B}|g(x)|$ due to ℬ being bounded.^a^ Secondly, the linear operator *K* is the integral operator defined by the kernel *w*

(3) $$Kg(x)=\int_{B}w(x,y)g(y)dy\;\forall g\in L^{2}(B).$$

Throughout the paper, we assume that 

(H2) the kernel *w* is such that *K* is a compact, self-adjoint operator on $L^{2}(B)$.

 We note that an integral operator is self-adjoint if and only if the kernel is symmetric, i.e., w(x,y)=w(y,x) for all x,y∈B. A sufficient condition for the compactness of *K* is, e.g., ${∥w∥}_{L^{2}(B\times B)}<\infty$ in which case the operator is called a Hilbert–Schmidt operator. Since ℬ is bounded, the continuity of the kernel *w* on B×B implies the compactness of *K* considered an integral operator on C(B).

Then we rewrite Eq. (2) as an Hilbert space-valued stochastic evolution equation

(4) $$dU_{t}=\left[-\alpha U_{t}+KF(U_{t})\right]dt+\epsilon dW_{t},$$

where *W* is an $L^{2}(B)$-valued stochastic process. Interpreting the original equation in this form, we now give a definition of the noise process assuming that 

(H3) *W* is a *Q*-Wiener process on $L^{2}(B)$, where the covariance operator *Q* is a nonnegative, symmetric trace class operator on $L^{2}(B)$.

 For a detailed explanation of a Hilbert space-valued *Q*-Wiener process and its covariance operator, we refer to, e.g., [23,56]. As the operator *Q* is nonnegative, symmetric, and of trace class there exists an orthonormal basis of $L^{2}(B)$ consisting of eigenfunctions $v_{i}$ and corresponding non-negative real eigenvalues $\lambda_{i}^{2}$, which satisfy $\sum_{i=1}^{\infty }\lambda_{i}^{2}<\infty$ . It then holds that the *Q*-Wiener process *W* satisfies

(5) $$W_{t}=\sum_{i=1}^{\infty }\lambda_{i}\beta_{t}^{i}v_{i},$$

where $\beta^{i}$ are a sequence of independent scalar Wiener processes (cf. [[56], Proposition 2.1.10]). The series (5) converges in the mean-square on $C([0,T],L^{2}(B))$. Furthermore, a straightforward adaptation of the proof of [[56], Proposition 2.1.10] shows that convergence in the mean-square also holds in the space C([0,T],C(B)) for every T>0 if $v_{i}\in C(B)$ for all *i* (corresponding to nonzero eigenvalues) and $\sup_{x\in B}|\sum_{i=1}^{\infty }\lambda_{i}^{2}v_{i}{\left(x\right)}^{2}|<\infty$.

The existence and uniqueness of mild solutions to (4) with trace class noise for given initial condition $U_{0}\in L^{2}(B)$ is guaranteed under the Lipschitz condition on *f*, cf. [23], and we can write the solution in its mild form

(6) $$U_{t}=e^{-\alpha t}U_{0}+\int_{0}^{t}e^{-\alpha (t-s)}KF(U_{s})ds+\int_{0}^{t}e^{-\alpha (t-s)}dW_{s}.$$

The solution possesses a modification in $C([0,T],L^{2}(B))$ and from now on we always identify the solution (6) with its continuous modification. It is worthwhile to note that for cylindrical Wiener processes—and thus in particular space-time white noise—there does not exist a solution to (4). This contrasts with other well-studied infinite-dimensional stochastic evolution equations, e.g., the stochastic heat equation. Due to the representation of the solution (6), it follows that a solution can only be as spatially regular as the stochastic convolution $\int_{0}^{t}e^{-\alpha (t-s)}dW_{s}$. In the present case, the semigroup generated by the linear operator is not smoothing in contrast to, e.g., the semigroup generated by the Laplacian in the heat equation. Thus, the stochastic convolution is only as smooth as the noise which for space-time white noise is not even a well-defined function. To be more specific, for cylindrical Wiener noise, the series representation of the stochastic convolution (cf. see Eq. (8) below) does not converge in a suitable probabilistic sense.

We next aim to strengthen the spatial regularity of the solution (6), which will be required later on. According to [[23], Theorem 7.10] the solution (6) is a continuous process taking values in the Banach space C(B) if the initial condition satisfies $u_{0}\in C(B)$, the linear part in the drift of (4) generates a strongly continuous semigroup on C(B), the nonlinear term *KF* is globally Lipschitz continuous on C(B), and finally, if the stochastic convolution is a continuous process taking values in C(B). It is easily seen that the first conditions are satisfied and sufficient conditions for the latter property are given in the following lemma.

**Lemma 2.1***Assume that the orthonormal basis functions*$v_{i}$*are Lipschitz continuous with Lipschitz constants*$L_{i}$*such that*

(7) $$\underset{x\in B}{\sup}|\sum_{i=1}^{\infty }\lambda_{i}^{2}v_{i}{\left(x\right)}^{2}|<\infty ,\;\;\underset{x\in B}{\sup}|\sum_{i=1}^{\infty }\lambda_{i}^{2}L_{i}^{2\rho }{\left|v_{i}(x)\right|}^{2(1-\rho )}|<\infty$$

*for a*ρ∈(0,1). *Then the process*

(8) $$O(x,t):=\int_{0}^{t}e^{-\alpha (t-s)}dW_{s}(x)=\sum_{i=1}^{\infty }\lambda_{i}\int_{0}^{t}e^{-\alpha (t-s)}d\beta_{s}^{i}v_{i}(x)$$

*possesses a modification withγ*-*Hölder continuous paths in*$R_{+}\times B$*for all*γ∈(0,ρ/2).

*Proof* We prove the lemma applying the Kolmogorov–Centsov theorem (cf. [[23], Theorem 3.3 and Theorem 3.4]). Throughout the proof, *C* is some finite constant, which may change from line to line, but is independent of x,y∈B and t,s≥0. We start showing that the process *O* is Hölder continuous in the mean-square in each direction. As $v_{i}$ are assumed continuous these are pointwise uniquely given and each O(t,x) is for fixed x∈B and t≥0 a Gaussian random variable due to $\sum_{i=1}^{\infty }\lambda_{i}^{2}v_{i}{\left(x\right)}^{2}<\infty$. Hence, for all 0≤s≤t and all x,y∈B, we obtain

$$\begin{array}{rcl} E{\left|O(x,t)-O(y,t)\right|}^{2} & = & \sum_{i=1}^{\infty }\lambda_{i}^{2}\int_{0}^{t}e^{-2\alpha (t-s)}ds{\left|v_{i}(x)-v_{i}(y)\right|}^{2}\\ & \leq & C\underset{z\in B}{\sup}|\sum_{i=1}^{\infty }\lambda_{i}^{2}L_{i}^{2\rho }{\left|v_{i}(z)\right|}^{2(1-\rho )}|{\left|x-y\right|}^{\rho } \end{array}$$

using

$${\left|v_{i}(x)-v_{i}(y)\right|}^{2}\leq L_{i}^{2\rho }{\left|x-y\right|}^{\rho }{\left|x-y\right|}^{\rho }{\left(\left|v_{i}(x)\right|+\left|v_{i}(y)\right|\right)}^{2(1-\rho )}$$

for every ρ∈[0,1] and ${\left|x-y\right|}^{\rho }\leq \mathrm{diam}{\left(B\right)}^{\rho }$. Next, for the temporal regularity we obtain

$$\begin{array}{rcl} & & E{\left|O(x,t)-O(x,s)\right|}^{2}\\ & & \;=\sum_{i=1}^{\infty }\lambda_{i}^{2}v_{i}{\left(x\right)}^{2}\int_{s}^{t}e^{-2\alpha (t-r)}dr+\sum_{i=1}^{\infty }\lambda_{i}^{2}v_{i}{\left(x\right)}^{2}\int_{0}^{s}|e^{-\alpha (t-r)}-e^{-\alpha (s-r)}{|}^{2}dr\\ & & \;=\sum_{i=1}^{\infty }\lambda_{i}^{2}v_{i}{\left(x\right)}^{2}\left(\frac{1-e^{-\alpha (t-s)}}{2\alpha }\right)\\ & & \;\;+\sum_{i=1}^{\infty }\lambda_{i}^{2}v_{i}{\left(x\right)}^{2}\left(\frac{{\left(1-e^{-\alpha (t-s)}\right)}^{2}-{\left(e^{-\alpha t}-e^{-\alpha s}\right)}^{2}}{2\alpha }\right). \end{array}$$

As the exponential function on the negative half-axis is Hölder continuous for every ρ∈[0,1], it holds

$$E{\left|O(x,t)-O(x,s)\right|}^{2}\leq C_{\rho }\sum_{i=1}^{\infty }\lambda_{i}^{2}v_{i}{\left(x\right)}^{2}{\left|t-s\right|}^{\rho }.$$

Thus, overall Jensen’s inequality yields $E{\left|O(x,t)-O(y,s)\right|}^{2}\leq C_{\rho }{\left({\left|x-y\right|}^{2}+{\left|t-s\right|}^{2}\right)}^{\rho /2}$. Since the difference O(x,t)−O(y,s) is centered Gaussian, it further holds that

$$E{\left|O(x,t)-O(y,s)\right|}^{2m}\leq C_{\rho ,m}{\left({\left|t-s\right|}^{2}+{\left|x-y\right|}^{2}\right)}^{m\rho /2}\;\forall m\in N.$$

Now, the Kolmogorov–Centsov theorem implies the statement of the lemma. □

We present an example to illustrate the type of noise we are generally interested in. Further motivation is provided in Sect. 3.

*Example 2.1* Consider the neural field equation on a *d*-dimensional cube $B={\left[0,2\pi \right]}^{d}$ with noise based on trigonometric basis functions of $L^{2}({\left[0,2\pi \right]}^{d})$. This type of noise is almost ubiquitous in applications as for the stochastic heat equations the basis functions can be chosen such that the usual (Dirichlet, Neumann or periodic) boundary conditions are preserved. For the example of noise preserving homogeneous Neumann boundary conditions, the basis functions are

(9) $$v_{i}(x)=\prod_{k=1}^{d}e_{i_{k}}(x_{k}),$$

where $x=(x_{1},\ldots ,x_{d})$, $i=(i_{1},\ldots ,i_{k})$ is a multi-index in $N^{d}$ and the functions $e_{i_{k}}$ are given by

$$e_{i_{k}}(x_{k})=\{\begin{array}{cc} \frac{1}{\sqrt{2\pi }}, & i_{k}=0,\\ \frac{1}{\sqrt{\pi }}\cos(i_{k}x_{k}/2), & i_{k}\geq 1. \end{array}$$

The functions $v_{i}$ are for all $i\in N^{d}$ pointwise bounded by $\pi^{-d/2}$ and Lipschitz continuous with Lipschitz constants given by $L_{i}=\pi^{-d/2}|i|$ (cf. [[8], Lemma 5.3]). Next, we construct a trace class Wiener process from these basis functions. A particular important example of spatiotemporal noise is smooth noise with exponentially decaying spatial correlation length [15,36,43], i.e.,

(10) $$\begin{array}{rcl} EW_{t}(x)W_{s}(y) & = & \min\{t,s\}\frac{1}{{\left(2\xi \right)}^{d}}\exp\left(-\frac{\pi }{4}\frac{{\left|x-y\right|}^{2}}{\xi^{2}}\right)\\ & & +\text{correction on the boundary} \end{array}$$

for a parameter ξ>0 modeling the spatial correlation length. Note that for ξ→0 this noise process approximates space-time white noise. Following [60], we can calculate under the assumption that ξ≪2π the coefficients $\lambda_{i}^{2}$ such that the *Q*-Wiener process (5) possesses the correlation function (10) and obtain

(11) $$\lambda_{i}^{2}=\exp\left(\frac{-\xi^{2}{\left|i\right|}^{2}}{4\pi }\right).$$

Now, it is easy to see that for this choice of eigenvalues the noise is of trace class and moreover the additional conditions of Lemma 2.1 are satisfied: As the functions $v_{i}$ are bounded, we obtain

$$\begin{array}{rcl} \underset{x\in B}{\sup}|\sum_{i\in N^{d}}^{\infty }\lambda_{i}^{2}v_{i}{\left(x\right)}^{2}| & \leq & \pi^{-d}+\pi^{-d}\sum_{N=1}^{\infty }\sum_{i\in {\left\{0,\ldots ,N\right\}}^{d}\setminus {\left\{0,\ldots ,N-1\right\}}^{d}}\exp\left(\frac{-\xi^{2}{\left|i\right|}^{2}}{4\pi }\right)\\ & \leq & \pi^{-d}+\pi^{-d}\sum_{N=0}^{\infty }\exp\left(\frac{-\xi^{2}N^{2}}{4\pi }\right)2^{N-1}\\ & < & \infty \end{array}$$

and the second condition of (7) is satisfied as

$$\begin{array}{rcl} \underset{x\in B}{\sup}|\sum_{i=N^{d}}\lambda_{i}^{2}L_{i}^{2\rho }{\left|v_{i}(x)\right|}^{2(1-\rho )}| & \leq & \pi^{-d}\sum_{N=1}^{\infty }\sum_{i\in {\left\{0,\ldots ,N\right\}}^{d}\setminus {\left\{0,\ldots ,N-1\right\}}^{d}}\exp\left(\frac{-\xi^{2}{\left|i\right|}^{2}}{4\pi }\right){\left|i\right|}^{2\rho }\\ & \leq & \pi^{-d}\sqrt{d}\sum_{N=0}^{\infty }\exp\left(\frac{-\xi^{2}N^{2}}{4\pi }\right)N^{2\rho }2^{N-1}\\ & < & \infty . \end{array}$$

## 3 Gain Function Perturbation

Another motivation for the considered additive noise neural field equations stems from a (formal) perturbation of the gain function *f* with space-time white noise. Let $\dot{W}$ denote space time white noise and consider the randomly perturbed Amari equation

(12) $$\partial_{t}U(x,t)=-\alpha U(x,t)+\int_{B}w(x,y)\left(f\left(U(y,t)\right)+\epsilon \dot{W}(y,t)\right)dy.$$

Recall that, by assumption (H2), the integral operator *K* defined by the kernel *w* is a self-adjoint compact operator. Thus, the spectral theorem implies that *K* possess only real eigenvalues $\lambda_{i}$, i∈N, and the corresponding eigenfunctions $v_{i}$ form an orthonormal basis of $L^{2}(B)$. If additionally we assume that 

(H4) *K* is a Hilbert–Schmidt operator on $L^{2}(B)$, that is, ${∥w∥}_{L^{2}(B\times B)}<\infty$,

 then the eigenvalues satisfy $\sum_{i=1}^{\infty }\lambda_{i}^{2}<\infty$. Hence, *K* possesses the series representation

$$Kg=\sum_{i=1}^{\infty }\lambda_{i}\langle g,v_{i}\rangle v_{i}\;\forall g\in L^{2}(B)$$

which yields for the perturbed equation (12) the representation

$$\partial_{t}U(x,t)=-\alpha U(x,t)+\sum_{i=1}^{\infty }\lambda_{i}\left(\int_{B}f\left(U(y,t)\right)v_{i}(y)dy+\epsilon \langle \dot{W}(t,\cdot ),v_{i}\rangle \right)v_{i}(x).$$

Next, note that the random variables ${\dot{\beta }}_{t}^{i}=\langle \dot{W}(\cdot ,t),v_{i}\rangle$ form a sequence of independent scalar white noise processes in time. Therefore, the perturbed equation becomes

$$\partial_{t}U(x,t)=-\alpha U(x,t)+\int_{B}w(x,y)f\left(U(y,t)\right)dy+\epsilon \sum_{i=1}^{\infty }\lambda_{i}{\dot{\beta }}_{t}^{i}v_{i}(x).$$

Rewriting this equation in the usual notation of stochastic differential equations we obtain

(13) $$dU_{t}(x)=\left[-\alpha U_{t}(x)+\int_{B}w(x,y)f\left(U_{t}(y)\right)dy\right]dt+\epsilon dW_{t}(x),$$

where

$$W_{t}(x)=\sum_{i=1}^{\infty }\lambda_{i}\beta_{t}^{i}v_{i}(x)$$

is a trace-class Wiener process on the Hilbert space $L^{2}(B)$. Note, when comparing to (5) here the coefficients $\lambda_{i}$ may be negative, however, as $-\beta^{i}$ is also a Wiener process this slight inconsistency can be neglected.

We next want to discuss spatial continuity of the solution to this equation with its particular noise structure. It is clear that this should translate into smoothing conditions of the kernel *w*. Due to Lemma 2.1, it is sufficient to establish conditions (7): First, it holds that

$$\sum_{i=1}^{\infty }\lambda_{i}^{2}v_{i}{\left(x\right)}^{2}=\sum_{i=1}^{\infty }{\left(\int_{B}w(x,y)v_{i}(y)dy\right)}^{2}=\sum_{i=1}^{\infty }{\langle w(x,\cdot ),v_{i}\rangle }^{2}={∥w(x,\cdot )∥}_{L^{2}(B)}^{2}$$

due to Parseval’s identity. Hence, the first condition of (7) becomes

(14) $$\underset{x\in B}{\sup}{∥w(x,\cdot )∥}_{L^{2}(B)}<\infty .$$

Next, the basis functions are continuous if the kernel w(x,y) is continuous in *x* and as the minimal Lipschitz constant is given by the supremum on the derivatives we obtain

$$L_{i}=\underset{x\in B}{\sup}|\frac{1}{\lambda_{i}}\nabla_{x}\int_{B}w(x,y)v_{i}(y)dy|\leq \frac{1}{\left|\lambda_{i}\right|}\underset{x\in B}{\sup}{∥\nabla_{x}w(x,\cdot )∥}_{L^{2}(B)}$$

due to the Cauchy–Schwarz inequality. Therefore, the second condition in (7) is satisfied if

(15) $$\begin{array}{rl} \underset{x\in B}{\sup}{∥\nabla_{x}w(x,\cdot )∥}_{L^{2}(B)} & <\infty \;\text{and}\\ \sum_{i=1}^{\infty }{\left|\lambda_{i}\right|}^{2(1-\rho )}{\left|v_{i}(x)\right|}^{2(1-\rho )} & \leq M\;\forall x\in B \end{array}$$

for a ρ∈(0,1) and a M<∞. The condition (14) and the first part of (15) are easily checked but for the second part of (15) usually theoretical results on the speed of decay of the eigenvalues have to be obtained. We note that (15) is certainly satisfied with ρ=1/2 if *K* is a trace class operator and the eigenfunctions are pointwise bounded independently of *i*; see, e.g., Example 2.1.

## 4 Deterministic Dynamics

The classical deterministic Amari model, obtained for ϵ=0 in (2), is

(16) $$\partial_{t}U(x,t)=-\alpha U(x,t)+\int_{B}w(x,y)f\left(U(y,t)\right)dy.$$

where $B\subseteq R^{d}$. Note that we may allow ℬ to be unbounded for the deterministic case as solutions of (16) do exist in this case [55]. Suppose there exists a stationary solution $U^{*}=U^{*}(x)$ of (16). To determine the stability of $U^{*}$ consider $U(x,t)=U^{*}(x)+\psi (x,t)$. Substituting into (16) and Taylor-expanding around $U^{*}$ yields the linearized problem

(17) $$\partial_{t}\psi (x,t)=-\alpha \psi (x,t)+\int_{B}w(x,y)(Df)\left(U^{*}(y)\right)\psi (y,t)dy.$$

Hence, the standard ansatz $\psi (x,t)=\psi_{0}(x)e^{\mu t}$ leads to the eigenvalue problem

(18) $$\begin{array}{rl} \underset{=:\eta }{\underset{︸}{\left(\mu +\alpha \right)}}\psi_{0}(x) & =\int_{B}w(x,y)(Df)\left(U^{*}(y)\right)\psi_{0}(y)dy:=(L\psi_{0})(x)\;\text{or}\\ L\psi_{0} & =\eta \psi_{0}. \end{array}$$

The linear stability condition μ<0 is equivalent to η<α where η∈spec(L). The stability analysis can be reduced to the understanding of the operator ℒ. However, this is a highly nontrivial problem as the behavior depends upon ℬ, $U^{*}(x)$, w(x,y), and f(u).

An LDP and Kramers’ law are of particular interest in the case of bistability. Therefore, we point out that there are many situations where (16) does have three stationary solutions: $U_{\pm }^{*}(x)$, which are stable and $U_{0}^{*}(x)$ which is unstable. The following three examples make this claim more precise.

*Example 4.1* The first example is presented by Ermentrout and McLeod [29]. Let B=R, w(x,y)=w(|x−y|), α=1 and assume that 0≤U(x,t)≤1. Furthermore, suppose that $f\in C^{1}([0,1],R)$ with $f^{\prime }>0$ and

(19) $$\tilde{f}(U):=-U+f(U)$$

has precisely three zeros U=0,a,1 with 0<a<1. The additional conditions $f^{\prime }(0)<1$ and $f^{\prime }(1)<1$ guarantee stability of the stationary solutions U=0 and U=1. As an even more explicit assumption [[29], p. 463], one may consider a Dirac *δ*-distribution for *w* in (16), which yields

(20) $$\partial_{t}U(x,t)=-U(x,t)+F\left(U(x,t)\right).$$

Suppose there are precisely three solutions for U=F(U) given by U=0,a,1 with 0<a<1. If $F^{\prime }(0)<1$, $F^{\prime }(1)<1$ and $F^{\prime }(a)>1$ then (20) has an unstable stationary solution between the two stable stationary solutions.

*Example 4.2* An even more concrete example is given by Guo and Chow [39,40]. They assume B=R, w(x,y)=w(x−y), α=1 and fix two functions

$$f(u)=\left[b(u-u_{b})+1\right]H(u-u_{b}),\;\;w(x)=Ae^{-a|x|}-e^{\left|x\right|}$$

where H(⋅) is the Heaviside function and *b*, *a*, *A*, and $u_{b}$ are parameters. Depending on parameter values, one may obtain three constant stationary solutions exhibiting bistability as expected from Example 4.1. However, there are also parameter values so that three stationary pulses exhibiting bistability exist.

Note that the choice B=R is not essential to obtain two deterministically-stable stationary states $U_{\pm }^{*}(x)$ and one deterministically-unstable stationary state $U_{0}^{*}(x)$. The important aspect is that certain algebraic equations, such as U=f(U) and U=F(U) in Example 4.1, have the correct number of solutions. Furthermore, one has to make sure that the sign of the nonlinearity *f* is chosen correctly to obtain the desired deterministic stability results for the stationary solutions. Hence, we expect that a similar situation also holds for bounded domains; see also [63]. 

Examples 4.1–4.2 are typical for many similar cases with x∈R or $x\in R^{2}$. Many results on existence and stability of stationary solutions are available; see, e.g., [1,46,51,52], and references therein. 

*Example 4.3* As a higher-dimensional example, one may consider the work by Jin, Liang, and Peng [42] who assume that w(x,y)=w(x−y), α=1, $B=R^{d}$, and

$$Z_{\infty }=\int_{R^{d}}w(x)dx<\infty ,\;\kappa Z_{\infty }>1,$$

where *κ* is the Lipschitz constant of $f\in C^{1}(R^{d},R)$. Furthermore, suppose $f^{\prime }$ is uniformly continuous and

$$\begin{array}{rcl} f^{\prime }(U)Z_{\infty } & < & 1\;\text{for }U\in (-\infty ,U_{1})\cup (U_{2},\infty ),\\ f^{\prime }(U)Z_{\infty } & = & 1\;\text{for }U\in \{U_{1},U_{2}\},\\ f^{\prime }(U)Z_{\infty } & > & 1\;\text{for }U\in (U_{1},U_{2}), \end{array}$$

for $U_{1}<0<U_{2}$. Then [[42], Proposition 11] the conditions

$$-U_{1}+f(U_{1})Z_{\infty }<0\;\text{and}\;-U_{2}+f(U_{2})Z_{\infty }>0$$

yield three stationary solutions $U_{+}^{*}$, $U_{-}^{*}$ and $U_{0}^{*}$. The solutions $U_{\pm }^{*}$ are stable and satisfy $U_{-}^{*}\leq 0$ and $U_{+}^{*}>0$. The solution $U_{0}^{*}$ is unstable.

Although we only focus on stationary solutions, it is important to remark that the techniques developed here could—in principle—also be applied to traveling waves U(x,t)=U(x−st) for s>0. The existence and stability of traveling waves for (16) has been investigated for many different situations; see, e.g. [12,14,21,29], and references therein. However, it seems reasonable to restrict ourselves here to the stationary case as even for this simpler case an LDP and Kramers’ law are not yet well understood. 

## 5 Large Deviations and Kramers’ Law

Here, we briefly introduce the background and notation for LDPs and Kramers’ law needed through the remaining part of the paper; see [26,34] for more details. Consider a topological space  with Borel *σ*-algebra $B_{X}$. A mapping I:X→[0,∞] is called a good rate function if it is lower semicontinuous and the level set {h:I(h)≤α} is compact for each α∈[0,∞). Sometimes the term action functional is used instead of rate function. Consider a family $\left\{\mu^{\epsilon }\right\}$ of probability measures on $\left(X,B_{X}\right)$. The measures $\left\{\mu^{\epsilon }\right\}$ satisfy an LDP with good rate function *I* if

(21) $$-\underset{\Gamma^{o}}{\inf}I\leq \underset{\epsilon \to 0}{lim inf}\epsilon^{2}\ln\mu^{\epsilon }(\Gamma )\leq \underset{\epsilon \to 0}{lim sup}\epsilon^{2}\ln\mu^{\epsilon }(\Gamma )\leq -\underset{\bar{\Gamma }}{\inf}I$$

holds for any measurable set Γ⊂X; often infima over the interior $\Gamma^{o}$ and closure $\bar{\Gamma }$ coincide so that lim inf and lim sup coincide at a common limit. One of the most classical cases is the application of (21) to finite-dimensional SDEs

(22) $$du_{t}=g(u_{t})dt+\epsilon G(u_{t})d\beta_{t}$$

where $u_{t}\in R^{N}$, $g:R^{N}\to R^{N}$, $G:R^{N}\to R^{N\times k}$, $\beta_{t}={\left(\beta_{t}^{1},\ldots ,\beta_{t}^{k}\right)}^{T}$ is a vector of *k* independent Brownian motions and we shall assume that the initial condition $u_{0}\in R^{N}$ is deterministic. If we want to emphasize that $u_{t}$ depends on *ϵ*, we shall also use the notation $u_{t}^{\epsilon }$. The topological space is chosen as a path space

$$X:=C_{0}\left([0,T],R^{N}\right)=\left\{\phi \in C\left([0,T],R^{N}\right):\phi (0)=u_{0}\right\}.$$

To state the next result, we also need the Sobolev space

(23) $$H_{1}^{N}:=\left\{\phi :[0,T]\to R^{N}:\phi \text{ absolutely continuous, }\phi^{\prime }\in L^{2},\phi (0)=0\right\}.$$

Furthermore, we are going to assume that the diffusion matrix $D(u):=G{\left(u\right)}^{T}G(u)\in R^{N\times N}$ is positive definite.

**Theorem 5.1** ([26,34]) 

*The SDE* (22) *satisfies the LDP* (21) *given by*

(24) $$\begin{array}{rcl} -\underset{\Gamma^{o}}{\inf}I & \leq & \underset{\epsilon \to 0}{lim inf}\epsilon^{2}\ln P\left({\left(u_{t}^{\epsilon }\right)}_{t\in [0,T]}\in \Gamma \right)\\ & \leq & \underset{\epsilon \to 0}{lim sup}\epsilon^{2}\ln P\left({\left(u_{t}^{\epsilon }\right)}_{t\in [0,T]}\in \Gamma \right)\leq -\underset{\bar{\Gamma }}{\inf}I \end{array}$$

*for any measurable set of paths*Γ⊂X*with good rate function*

(25) $$\begin{array}{rcl} I(\phi ) & = & I_{\left[0,T\right]}(\phi )\\ & = & \left\{ \begin{array}{cc} \frac{1}{2}\int_{0}^{T}{\left(\phi_{t}^{\prime }-g(\phi_{t})\right)}^{T}D{\left(\phi_{t}\right)}^{-1}(\phi_{t}^{\prime }-g(\phi_{t}))dt, & \text{if }\phi \in u_{0}+H_{1}^{N},\\ +\infty , & \text{otherwise.} \end{array}\right. \end{array}$$

An important application of the LDP (24) is the so-called first-exit problem. Suppose that $u_{t}$ starts near a stable equilibrium $u^{*}\in D\subset R^{N}$ of the deterministic system given by setting ϵ=0 in (22), where  is a bounded domain with smooth boundary. Define the first-exit time

(26) $$\tau_{D}^{\epsilon }:=\inf\left\{t>0:u_{t}^{\epsilon }\notin D\right\}.$$

To formalize the application of the LDP, define the mapping

(27) $$Z(u,v;s):=\inf\left\{I(\phi ):\phi \in C\left([0,s],R^{N}\right),\phi_{0}=u,\phi_{s}=v\right\}$$

which is the cost for a path starting at *u* to reach *v* in time *s*. Next, assume that $\bar{D}$ is properly contained inside the (deterministic) basin of attraction of $u^{*}$. Then one can show [[34], Theorem 4.1, p. 124] that

(28) $$\lim_{\epsilon \to 0}\epsilon^{2}\ln P\left(\tau_{D}^{\epsilon }\leq t|u=u_{0}\right)=\inf\left\{Z(u,v;s):s\in [0,t],v\notin D\right\}.$$

To get more precise information on the exit distribution, one defines the function

$$Z\left(u^{*},v\right)=\underset{t>0}{\inf}Z\left(u^{*},v;t\right)$$

which is called the quasipotential for $u^{*}$. It is natural to minimize the quasipotential over ∂D and define

$$\bar{Z}:=\underset{v\in \partial D}{\inf}Z\left(u^{*},v\right).$$

**Theorem 5.2** ([[34], Theorem 4.2, p. 127], [[26], Theorem 5.7.11]) 

*For all initial conditions*u∈D*and all*δ>0, *the following two limits hold*:

(29) $$\lim_{\epsilon \to 0}P\left(e^{(\bar{Z}-\delta )/\epsilon^{2}}<\tau_{D}^{\epsilon }<e^{(\bar{Z}+\delta )/\epsilon^{2}}|u_{0}=u^{*}\right)=1,$$

(30) $$\lim_{\epsilon \to 0}\epsilon^{2}\ln E\left[\tau_{D}^{\epsilon }|u_{0}=u^{*}\right]=\bar{Z}.$$

If the SDE (22) has a gradient structure with identity diffusion matrix, i.e.,

(31) $$g(u)=-\nabla V(u)\;\text{for }V:R^{N}\to R,\;\text{and}\;G(u)=\mathrm{Id}\in R^{N\times N}$$

then one can show [[34], Sect. 4.3] that the quasipotential is given by $Z(u^{*},v)=2(V(v)-V(u^{*}))$. If the potential has precisely two local minima $u_{\pm }^{*}$ and a saddle point $u_{s}^{*}$ with N−1 stable directions so that the Hessian $\nabla^{2}V(u_{s}^{*})$ has eigenvalues

$$\rho_{1}\left(u_{s}^{*}\right)<0<\rho_{2}\left(u_{s}^{*}\right)<\cdots <\rho_{N}\left(u_{s}^{*}\right)$$

then one can even refine Theorem 5.2. Suppose $u_{0}=u_{-}^{*}$ then the mean first passage time to $u_{+}^{*}$ satisfies

(32) $$\begin{array}{rcl} & & E\left[\inf\left\{t>0:{∥u_{t}-u_{+}^{*}∥}_{2}\leq \delta \right\}\right]\\ & & \;∼\frac{2\pi }{\left|\rho_{1}(u_{s}^{*})\right|}\sqrt{\frac{\left|\det(\nabla^{2}V(u_{s}^{*}))\right|}{\det(\nabla^{2}V(u_{-}^{*}))}}e^{2(V(u_{s}^{*})-V(u_{-}^{*}))/\epsilon^{2}} \end{array}$$

where ${∥\cdot ∥}_{2}$ denotes the usual Euclidean norm in $R^{N}$. The formula (32) is also known as Kramers’ law [5] or Arrhenius–Eyring–Kramers’ law [2,31,45]. Note that the key differences with the general LDP (29) for the first-exit problem are that (32) yields a precise prefactor for the exponential transition time and uses the explicit form of the good rate function for gradient systems. It is interesting to note that a rigorous proof of (32) has only been obtained quite recently [9,10]. 

## 6 Gradient Structures in Infinite Dimensions

The finite-dimensional Kramers’ formula (32) applies to SDEs (22) with a gradient-structure g(u)=−∇V(u) where $V:R^{N}\to R$ is the potential. A generalization of Kramers’ law has been carried over to the infinite-dimensional case of SPDEs given by

(33) $$dU=\left[\Delta U-h^{\prime }(U)\right]dt+\epsilon dW(x,t)$$

for U=U(x,t), $x\in \tilde{B}\subset R$, $\tilde{B}$ a bounded interval, $h\in C^{k}(R,R)$ for suitably large k∈N and W(x,t) denotes space-time white noise and either Dirichlet or Neumann boundary conditions are used [4,6,7]. A crucial reason why this generalization works is that the SPDE (33) has a gradient-type structure [32] given by the energy functional

(34) $$V[U]:=\int_{\tilde{B}}\left[\frac{1}{2}U^{\prime }{\left(x\right)}^{2}+h\left(U(x)\right)\right]dx.$$

More precisely, when ϵ=0 one obtains from (33) a PDE, say with Dirichlet boundary conditions,

(35) $$dU=\left[\Delta U-h^{\prime }(U)\right]dt,\;\;U(x)=0\;\text{on }\partial \tilde{B}$$

for a given sufficiently smooth initial condition $U(x,0)=U_{0}(x)\in C^{k}(R,R)$. Standard parabolic regularity [[30], Sect. 7.1] implies that solutions *U* of (35) lie in the Sobolev spaces $H_{0}^{k}(\tilde{B})$. Computing the Gâteaux derivative in this space yields

(36) $$\nabla_{z}V[U]=\int_{\tilde{B}}\left[-U^{\prime \prime }(x)+h^{\prime }\left(U(x)\right)\right]z(x)dx.$$

The Gâteaux derivative is equal to the Fréchet derivative ∇V=DV by a standard continuity result [[25], p. 47]. Hence, (36) shows that the stationary solutions of (35) are critical points of the gradient functional *V*. Since the gradient structure of the deterministic PDE (35) is a key structure to obtain a Kramers’-type estimate for the SPDE (33), we would like to check whether there is an analogue available for the deterministic Amari model (16).

We shall assume for simplicity that $f\in {\mathrm{BC}}^{1}(R)$ for the calculations in this section. Although this is a slightly stronger assumption than (H1), we shall see below that even with this assumption we are not able to obtain an immediate generalization of (36). Using a direct modification of the results in [55], it follows that the deterministic Amari model (16) has solutions U(x,t) in the Hölder space ${\mathrm{BC}}^{\alpha }(B)\times {\mathrm{BC}}^{\alpha }([0,T])$ for α∈(0,1] and $B\subset R^{d}$ is the usual domain we use for the Amari model. Now consider the analogous naive guess to (36) given by

(37) $$V[U]:=\int_{B}\left[\frac{\alpha }{2}U{\left(x\right)}^{2}-\int_{B}\int_{0}^{U(y)}f(r)w(x,y)drdy\right]dx.$$

Computing the derivative in ${\mathrm{BC}}^{\alpha }(B)$ yields

(38) $$\begin{array}{rcl} \nabla_{z}V[U] & = & \lim_{\delta \to 0}\frac{1}{\delta }\left(V[U+\delta z]-V[U]\right),\\ & = & \int_{B}\left[\alpha U(x)z(x)-\int_{B}f\left(U(y)\right)w(x,y)z(y)dy\right]dx. \end{array}$$

Therefore, setting $\nabla_{z}V[U]=0$ is not equivalent to the solution of the stationary problem

$$-\alpha U(x)+\int_{B}w(x,y)f\left(U(y)\right)dy=0.$$

Due to the presence of the different terms z(x) and z(y) in (38), one may guess that the modified functional

(39) $$V[U]:=\int_{B}\left[\frac{\alpha }{2}U{\left(x\right)}^{2}-\frac{1}{2}\int_{B}f\left(U(y)\right)f\left(U(x)\right)w(x,y)dy\right]dx$$

could work. However, another direct computation shows that

$$\begin{array}{rcl} \nabla_{z}V[U] & = & \int_{B}\left[\alpha U(x)z(x)dx\right]-\frac{1}{2}\left[\int_{B}\int_{B}f\left(U(x)\right)Df\left(U(y)\right)w(x,y)z(y)dydx\right]\\ & & -\frac{1}{2}\left[\int_{B}\int_{B}f\left(U(y)\right)Df\left(U(x)\right)w(x,y)z(x)dydx\right]\\ & = & \alpha \langle U,z\rangle -\langle KF(U),Df(U)z\rangle . \end{array}$$

Hence, *f* and its derivative *Df* both appear instead of the desired formulation; by a similar computation one can show that replacing f(u(⋅)) in (39) by $\int_{0}^{u}f(r)dr$ fails as well. Hence, there does not seem to be a natural generalization for the guess for the gradient functional (34). However, one has to consider possible coordinate changes. The idea to apply a preliminary transformation has been discussed, e.g., in [[28], p. 2] and [[51], p. 488]. Assume that

(40) $$f^{-1}=:g\text{ exists}\;\text{and}\;g^{\prime }\neq 0.$$

Define P(x,t):=f(U(x,t)) as the mean action-potential generating rate so that U=g(P). Observe that

(41) $$\partial_{t}P(y,t)=\frac{1}{g^{\prime }(P(x,t))}\left[-\alpha g\left(P(x,t)\right)+\int_{B}w(x,y)P(y,t)dy\right].$$

For this equation, the problem observed in (39) should disappear as the integral only contains linear terms. One may define an energy-type functional

$$E[P]:=\int_{B}\left[\int_{0}^{P(x)}\alpha g(r)dr-\frac{1}{2}\int_{B}w(x,y)P(y)P(x)dy\right]dx.$$

Calculating the derivative yields

$$\begin{array}{rcl} \nabla_{Q}E[P] & = & \lim_{\delta \to 0}\frac{1}{\delta }\left(E[P+\delta Q]-E[P]\right)\\ & = & \int_{B}\alpha g\left(P(x)\right)Q(x)dx\\ & & -\frac{1}{2}\int_{B}\int_{B}w(x,y)\left\{P(y)Q(x)+P(x)Q(y)\right\}dydx\\ & = & \langle \alpha g(P),Q\rangle -\langle \int_{B}w(x,y)P(y)dy,Q\rangle . \end{array}$$

This shows that there is hidden energy-type flow structure in the Amari model for the assumptions (40) so that

(42) $$\partial_{t}P(x,t)=-\frac{1}{g^{\prime }(P(x,t))}\nabla E\left[P(x,t)\right].$$

However, even with this variable transformation, there seems to be little hope to derive a precise Kramers’ rule for the stochastic Amari model (2) by generalizing the approach for SPDE systems [4,6,7]. The problems are as follows: 

– There is still a space-time dependent nonlinear prefactor $1/g^{\prime }(P(x,t))$ in (42) for the deterministic system, so the system is not an exact gradient flow for a potential.

– Applying the change-of-variable $P_{t}(x):=f(U_{t}(x))$ for the stochastic Amari model (2) requires an Itô-type formula so that

(43) $$\begin{array}{rcl} dP_{t}(x) & = & \frac{1}{g^{\prime }(P_{t}(x))}\left[-\alpha g\left(P_{t}(x)\right)+\int_{B}w(x,y)P_{t}(y)dy+O\left(\epsilon^{2}\right)\right]dt\\ & & +\epsilon M\left(P_{t}(x)\right)dW_{t}(x), \end{array}$$

where $M(P_{t}(x))$ is now a multiplicative noise term; see [24], and references therein for more details on infinite-dimensional Itô-type formulas. The higher-order term $O(\epsilon^{2})$ in the drift part of (43) is not expected to cause difficulties but a multiplicative noise structure definitely excludes the direct application of Kramers’ law.

– Even if we would just assume—without any immediate physical motivation—that the noise term in (43) is purely additive $\epsilon dW_{t}(x)$, there is a problem to apply Kramers’ law since we do not have a structure like in (22) with G(⋅)=Id as $W_{t}(x)$ is a *Q*-Wiener process defined in (5) and driving space-time white noise in (4) is particularly excluded due to the nonexistence of a solution.

Based on these observations, an immediate approach to generalize a sharp Kramers’ formula to neural fields seems unlikely. Hence we try to understand an LDP for the stochastic Amari-type model (2).

## 7 Direct Approach to an LDP

A general direct approach for the derivation of an LDP for infinite-dimensional stochastic evolution equations is presented in [23] and further results have been obtained for certain additional classes of SPDEs [17-19,57]. The results in [23] are valid for semilinear equations with suitable Lipschitz assumptions on the nonlinearity and with solutions taking values in C(D). We state the available results applied to continuous solutions of the Amari equation (4) assuming that the conditions of Lemma 2.1 are satisfied.

For the following, we assume that there exists an open neighborhood D∈C(B) containing a stable equilibrium state $u^{*}$ of the deterministic Amari equation (16) such that $\bar{D}$ is contained in the basin of attraction of $u^{*}$. We are interested in the rate function and the first-exit time of the process from  given by

$$\tau_{D}^{\epsilon }=\inf\{t\geq 0:U\notin D\}$$

if *U* starts in the deterministic equilibrium state $u^{*}$. In order to state the quasipotential for *u*, we consider the control system

(44) $$\dot{y}=-\alpha y+KF(y)+Q^{1/2}v,\;\;y_{0}=x\in C(B)$$

for controls $v\in L^{2}((0,T),L^{2}(B))$ for all T>0 and denote by $y^{x,v}$ its unique mild solution^b^ taking values in C([0,T],C(B)) for all T>0. Then we define

(45) $$I\left(u^{*},z\right)=\inf\left\{\frac{1}{2}\int_{0}^{T}{∥v(s)∥}_{L^{2}}^{2}ds:y^{u^{*},v}(T)=z,T>0\right\},$$

where this quasipotential relates to the minimal energy necessary to move the control system (44) started at the equilibrium state $u^{*}$ to *z*.

**Theorem 7.1** ([[23], Theorem 12.18]) 

It holds that

$$\lim_{\epsilon \to 0}\epsilon^{2}\ln E\left[\tau_{D}^{\epsilon }|U_{0}=u^{*}\right]=\underset{z\in \partial D}{\inf}I\left(u^{*},z\right).$$

Following further the exposition in [[23], Sect. 12] explicit formulae for the rate function *I* are only available in the special case of the drift possessing gradient structure and space-time white noise. As we have argued above, this structure is particularly not satisfied for neural field equations. Hence, the same observations as presented at the end of the last section prevent a further direct analytic approach to the LDP. Therefore, we try to understand the LDP problem for a discretized approximate finite-dimensional version of the neural field equation.

## 8 Galerkin Approximation

Throughout the section, we assume that the assumptions (H1)–(H3) are satisfied. As a discretized version of the neural field equation (2), we consider its spectral Galerkin approximations; recall that the solution $U_{t}$ of (2) lies in $C([0,T],L^{2}(B))$ as discussed in Sect. 2. In order to decouple the noise, we define the spectral representation of the solution

(46) $$U_{t}(x)=\sum_{i=1}^{\infty }u_{t}^{i}v_{i}(x).$$

Here, the orthonormal basis functions $v_{i}$ are given by the eigenfunctions of the covariance operator of the noise with corresponding eigenvalues $\lambda_{i}^{2}$, see Eq. (5). To obtain a equation for the coefficients $u_{t}^{i}$, we take the inner product of Eq. (4) with the basis functions $v_{i}$, which yields

$$\langle dU_{t},v_{i}\rangle =\left[-\alpha \langle U_{t},v_{i}\rangle +\langle KF(U_{t}),v_{i}\rangle \right]dt+\epsilon \langle dW_{t},v_{i}\rangle \;\text{for }i\in N.$$

After plugging in (46), we obtain for $u^{i}$ the countable Galerkin system

(47) $$du_{t}^{i}=\left[-\alpha u_{t}^{i}+{\left(KF\right)}^{i}\left(u_{t}^{1},u_{t}^{2},\ldots \right)\right]dt+\epsilon \lambda_{i}d\beta_{t}^{i}\;\text{for }i\in N.$$

Here, the nonlinearities coupling all the equations are given by

$$\begin{array}{rcl} {\left(KF\right)}^{i}\left(u_{t}^{1},u_{t}^{2},\ldots \right) & := & \int_{B}v_{i}(x)\left(\int_{B}w(x,y)f\left(\sum_{j=1}^{\infty }u_{t}^{j}v_{j}(y)\right)dy\right)dx\\ & = & \int_{B}f\left(\sum_{j=1}^{\infty }u_{t}^{j}v_{j}(x)\right)\left(\int_{B}w(x,y)v_{i}(y)dy\right)dx \end{array}$$

due to the symmetry of the kernel *w*. If, in addition, we assume that (H4) holds and *K* and *Q* possess the same eigenfunctions and the eigenvalues are related as discussed in Sect. 3 the nonlinearities become

(48) $${\left(KF\right)}^{i}\left(u_{t}^{1},u_{t}^{2},\ldots \right)=\lambda_{i}\int_{B}f\left(\sum_{j=1}^{\infty }u_{t}^{j}v_{j}(x)\right)v_{i}(x)dx.$$

The *N*th Galerkin approximation $U^{N}$ to *U* is obtained truncating the spectral representation (46), and thus given by

(49) $$U_{t}^{N}=\sum_{i=1}^{N}u_{t}^{i,N}v_{i},$$

where $u_{t}^{i,N}$ are the solutions to the *N*-dimensional Galerkin SDE system

(50) $$du_{t}^{i,N}=\left[-\alpha u_{t}^{i,N}+{\left(KF\right)}^{i,N}\left(u_{t}^{1,N},\ldots ,u_{t}^{N,N}\right)\right]dt+\epsilon \lambda_{i}d\beta_{t}^{i}\;\forall i=1,\ldots ,N,$$

where the nonlinearities $KF^{i,N}$ are given by

(51) $${\left(KF\right)}^{i,N}\left(u^{1,N},\ldots ,u^{N,N}\right)=\int_{B}f\left(\sum_{j=1}^{N}u^{j,N}v_{j}(x)\right)\left(\int_{B}w(x,y)v_{i}(y)dy\right)dx$$

or, in the special case of Sect. 3, by

(52) $${\left(KF\right)}^{i,N}\left(u^{1,N},\ldots ,u^{N,N}\right)=\lambda_{i}\int_{B}f\left(\sum_{j=1}^{N}u^{j,N}v_{j}(x)\right)v_{i}(x)dx,$$

respectively. The following theorem establishes the almost sure convergence of the Galerkin approximations to the solution of (4). Therefore, we may be able to infer properties of the behavior of paths of the solution from the path behavior of the Galerkin approximations. We have deferred the proof of the theorem to the Appendix.

**Theorem 8.1***It holds for all*T>0*that*

$$\lim_{N\to \infty }\underset{t\in [0,T]}{\sup}{∥U_{t}-U_{t}^{N}∥}_{L^{2}(B)}=0\;\text{a.s}.$$

*If*, *in addition*, *the series*$\sum_{i=1}^{\infty }\lambda_{i}^{2}v_{i}^{2}$*converges in*C(B)*and the functions*$v_{i}$*are Lipschitz continuous with Lipschitz constants*$L_{i}$*such that*$\sup_{x\in B}\sum_{i=1}^{\infty }\lambda_{i}^{2}L_{i}^{2\rho }{\left|v_{i}(x)\right|}^{2(1-\rho )}<\infty$*for a*ρ∈(0,1) (*i*.*e*., *the conditions of Lemma *2.1 *are satisfied*), $U_{0}\in C(B)$*such that*$\lim_{N\to \infty }{∥U_{0}-P^{N}U_{0}∥}_{0}=0$*andKis compact on*C(B), *then it holds for all*T>0*that*

$$\lim_{N\to \infty }\underset{t\in [0,T]}{\sup}{∥U_{t}-U_{t}^{N}∥}_{0}=0\;\text{a.s}.$$

## 9 Approximating the LDP

The LDP in Theorem 7.1 is not immediately computable. Here, we show that a finite-dimensional approximation can be made and what the structure of this approximation entails. For simplicity, consider the case when the diagonal diffusion matrix  with entries $D_{ii}=\lambda_{i}^{2}$ is positive definite, i.e., $\lambda_{i}\neq 0$ for all i∈N. Observe that the inverse of  induces an inner product on $R^{N}$ for N∈N∪{∞} via

$${\langle a,b\rangle }_{N}:=a^{T}{\left(D\right)}^{-1}b={\left[D^{-1/2}a\right]}^{T}\left[D^{-1/2}b\right]\;\text{for }a,b\in R^{N},$$

where  is understood as the projection onto $R^{N\times N}$ if N<∞. We are also going to use the notation introduced in Sect. 8 for the Galerkin approximation, i.e., $u_{t}^{\cdot ,N}$ denotes the vector

(53) $${\left(u_{t}^{1,N},u_{t}^{2,N},\ldots ,u_{t}^{N,N}\right)}^{T}\in R^{N}$$

where $u_{t}^{\cdot ,N}$ denotes the solutions of the *N*-dimensional system (50). Note that throughout this section we shall always work with the Galerkin coefficients, e.g., $u_{t}$ refers to the vector

$${\left(u_{t}^{1},u_{t}^{2},\ldots \right)}^{T}\in R^{\infty }.$$

Furthermore, for arbitrary functions $\phi_{t}\in L^{2}(B)$, which are used in the formulation of the rate function, we use the notation $\phi_{t}^{\cdot ,N}$ to denote the projection onto the first *N* Galerkin coefficients. Theorem 5.1 immediately implies the following:

**Proposition 9.1***For the finite*-*dimensional Galerkin system* (50) *the rate function is given by*

(54) IN(ϕ⋅,N)={12∫0T〈(ϕt⋅,N)′−g⋅,N(ϕt⋅,N),(ϕt⋅,N)′−g⋅,N(ϕt⋅,N)〉Ndt,+∞,if ϕ∈u0⋅,N+H1N,+∞,otherwise,

*where*$g^{i,N}(\phi_{t}^{\cdot ,N})=-\alpha \phi_{t}^{i,N}+{\left(KF\right)}^{i,N}(\phi_{t}^{1,N},\ldots ,\phi_{t}^{N,N})$.

Recall from Sect. 7 that Theorem 7.1 provides a large deviation principle. For the case when *Q* is a positive operator, we may formally rewrite the control system (44) as

(55) $$D^{-1/2}\left[\dot{y}-\left(-\alpha y+KF(y)\right)\right]=v$$

so that the rate function for the Amari model can be expressed as

(56) I(ϕ)={12∫0T∫BD−1/2[ϕt′−g(ϕt)]D−1/2[ϕt′−g(ϕt)]dxdt,+∞,if ϕ∈u0+H1∞,+∞,otherwise,

where $g(\phi_{t})=-\alpha \phi_{t}+KF(\phi_{t})$ and $D^{-1/2}u=\sum_{i=1}^{\infty }(D^{-1/2}u,v_{i})v_{i}$. Therefore, the next result just implies that the Galerkin approximation is consistent for the LDP.

**Proposition 9.2***For each*$\phi_{t}\in u_{0}+H_{1}^{\infty }$*we have*$\lim_{N\to \infty }|I(\phi_{t})-I^{N}(\phi_{t}^{\cdot ,N})|=0$.

*Proof* Considering the finite-dimensional rate function (54) it suffices to notice that

$$\begin{array}{rcl} & & {\langle {\left(\phi_{t}^{\cdot ,N}\right)}^{\prime }-g^{\cdot ,N}\left(\phi_{t}^{\cdot ,N}\right),{\left(\phi_{t}^{\cdot ,N}\right)}^{\prime }-g^{\cdot ,N}\left(\phi_{t}^{\cdot ,N}\right)\rangle }_{N}\\ & & \;=\sum_{i=1}^{N}\frac{1}{\lambda_{i}^{2}}{\left[{\left(\phi_{t}^{i,N}\right)}^{\prime }-g^{i,N}\left(\phi_{t}^{\cdot ,N}\right)\right]}^{2}\\ & & \;=\sum_{i=1}^{N}\int_{B}\frac{1}{\lambda_{i}^{2}}{\left[{\left(\phi_{t}^{i,N}v_{i}(x)\right)}^{\prime }-g^{i,N}\left(\phi_{t}^{\cdot ,N}\right)v_{i}(x)\right]}^{2}dx \end{array}$$

by orthonormality of the basis in $L^{2}(B)$. □

Hence, we may work with the finite-dimensional Galerkin system and its LDP for computational purposes. However, the truncation *N* may still be very large. We are going to show, using a formal analysis for a certain case, that there is an intrinsic multiscale structure of the rate function. We assume that we are in the special case considered in Sect. 3 where *K* and *Q* have the same eigenfunctions and the corresponding eigenvalues are given by $\lambda_{i}$ and $\lambda_{i}^{2}$, respectively.

**Lemma 9.1***For each*N∈N, *the first part of the rate function* (54) *can be rewritten as*

(57) $$I^{N}\left(\phi^{\cdot ,N}\right)=\frac{1}{2}\int_{0}^{T}a_{1}-2a_{2}+a_{3}dt$$

where the three terms are given by

$$\begin{array}{rcl} a_{1}^{N} & = & {\langle {\left(\phi_{t}^{\cdot ,N}\right)}^{\prime }+\alpha \phi_{t}^{\cdot ,N},{\left(\phi_{t}^{\cdot ,N}\right)}^{\prime }+\alpha \phi_{t}^{\cdot ,N}\rangle }_{N},\\ a_{2}^{N} & = & {\langle {\left(\phi_{t}^{\cdot ,N}\right)}^{\prime }+\alpha \phi_{t}^{\cdot ,N},KF^{\cdot ,N}\left(\phi_{t}^{\cdot ,N}\right)\rangle }_{N},\\ a_{3}^{N} & = & {\left[{\tilde{KF}}^{\cdot ,N}\left(\phi^{\cdot ,N}\right)\right]}^{T}\left[{\tilde{KF}}^{\cdot ,N}\left(\phi^{\cdot ,N}\right)\right] \end{array}$$

*and*${\left(\tilde{KF}\right)}^{i,N}=\frac{1}{\lambda_{i}^{2}}{\left(KF\right)}^{i,N}$.

*Proof* For notational simplicity, we shall temporarily omit in this proof the subscript for the inner product ${\langle \cdot ,\cdot \rangle }_{N}=\langle \cdot ,\cdot \rangle$ as well as the Galerkin index, e.g., $\phi_{t}^{\cdot ,N}=\phi_{t}$ as it is understood that we work with *N*-dimensional vectors in this proof. Consider the following general calculation:

$$\begin{array}{rcl} & & \langle \phi_{t}^{\prime }-g(\phi_{t}),\phi_{t}^{\prime }-g(\phi_{t})\rangle \\ & & \;=\langle \phi_{t}^{\prime },\phi_{t}^{\prime }\rangle -2\langle \phi_{t}^{\prime },g(\phi_{t})\rangle +\langle g(\phi_{t}),g(\phi_{t})\rangle \\ & & \;=\langle \phi_{t}^{\prime },\phi_{t}^{\prime }\rangle +2\alpha \langle \phi_{t}^{\prime },\phi_{t}\rangle -2\langle \phi_{t}^{\prime },KF(\phi_{t})\rangle \\ & & \;\;+\langle KF(\phi_{t}),KF(\phi_{t})\rangle +\alpha^{2}\langle \phi_{t},\phi_{t}\rangle -2\alpha \langle \phi_{t},KF(\phi_{t})\rangle \\ & & \;=\langle \phi_{t}^{\prime }+\alpha \phi_{t},\phi_{t}^{\prime }+\alpha \phi_{t}\rangle -2\langle \phi_{t}^{\prime }+\alpha \phi_{t},KF(\phi_{t})\rangle +\tilde{KF}{\left(\phi_{t}\right)}^{T}\tilde{KF}(\phi_{t}) \end{array}$$

and observe that the result is independent of *N*. □

It is important to point out that the LDP from Theorem 5.1 requires the infimum of the rate function. From Lemma 9.1, we know that the rate function splits into three terms. The three terms are interesting in the asymptotic limit N→∞. Suppose

$${\left(\phi_{t}^{\cdot ,N}\right)}^{\prime }+\alpha \phi_{t}^{\cdot ,N}=O\left(\kappa (N)\right)\;\text{and}\;{\tilde{KF}}^{\cdot ,N}\left(\phi_{t}^{\cdot ,N}\right)=O\left(\eta (N)\right)$$

as N→∞ for some nonnegative functions *κ*, *η*. Then Lemma 9.1 yields

$$a_{1}^{N}=O\left(\kappa {\left(N\right)}^{2}\lambda_{N}^{-2}\right),\;\;a_{2}^{N}=O\left(\kappa (N)\eta (N)\lambda_{N}^{-1}\right),\;\;a_{3}^{N}=O\left(\eta {\left(N\right)}^{2}\right).$$

**Lemma 9.2***Suppose there exists a positive constant*$K_{f}$*such that*

(58) $$\underset{x\in R}{\sup}\left|f(x)\right|\leq K_{f}$$

*then*η(N)=1.

*Proof* A direct estimate yields

$$|{\tilde{KF}}^{j,N}\left(\phi_{t}^{\cdot ,N}\right)|\leq \int_{B}|f\left(\sum_{i=1}^{N}\phi_{t}^{i,N}v_{i}(x)\right)|\left|v_{j}(x)\right|dx\leq K_{f}\int_{B}\left|v_{j}(x)\right|dx.$$

Since ${∥v_{j}∥}_{L^{2}(B)}=1$ and $L^{2}(B)↪L^{1}(B)$, the last integral is uniformly bounded over j∈N by $\mathrm{meas}{\left(B\right)}^{1/2}$. □

We remark that several typical functions *f* discussed in Sect. 2 such as $f(u)={\left(1+e^{-u}\right)}^{-1}$ and f(u)=(tanh(u)+1)/2 are globally bounded so that Lemma 9.2 does apply to many practical cases. In this situation, we get that

$$a_{1}^{N}=O\left(\kappa {\left(N\right)}^{2}\lambda_{N}^{-2}\right),\;\;a_{2}^{N}=O\left(\kappa (N)\lambda_{N}^{-1}\right),\;\;a_{3}^{N}=O(1).$$

We make a case distinction between the different relative asymptotics of κ(N) and $\lambda_{N}$. Note that the following asymptotic relations are purely formal: 

– If $\kappa (N)\ll \lambda_{N}$ or $\kappa (N)∼\lambda_{N}$ as N→∞, then we can conclude that κ(N)→0, i.e.,

(59) $${\left(\phi_{t}^{N,N}\right)}^{\prime }+\alpha \phi_{t}^{N,N}\to 0\;\text{as }N\to 0$$

since for trace-class noise we know that $\lambda_{N}\to 0$. If we formally require that ${\left(\phi_{t}^{N,N}\right)}^{\prime }+\alpha \phi_{t}^{N,N}=0$ for *N* sufficiently large, then the higher-order Galerkin modes decays exponentially in time

$$\phi_{t}^{N,N}=\phi_{0}^{N,N}e^{-\alpha t}.$$

– If $\kappa (N)\gg \lambda_{N}$ as N→∞, then $a_{1}\gg -2a_{2}+a_{3}$ and the first term dominates the asymptotics. But $a_{1}^{N}\geq 0$ for all *N* so that the rate function only has a finite infimum if $a_{1}^{N}\to 0$ as N→∞. This implies again that (59) holds for the case of a finite infimum.

Hence, we get in many reasonable first-exit problems for the Amari model with trace-class noise that there is a finite set for n≤N of “slow” or “center-like” directions and an infinite set of “fast” or “stable” directions for n>N. Although we have made this observation from the rate function alone, it is entirely natural considering the structure of the Galerkin approximation. Indeed, for the case when the eigenvalues of *K* and *Q* are related, we may write (50) as

(60) $$du_{t}^{i,N}=\left(-\alpha u_{t}^{i,N}+\lambda_{i}[\cdots ]\right)dt+\epsilon \lambda_{i}d\beta_{t}^{i}$$

so that for bounded nonlinearity *f*, which is represented in the terms [⋯] in (60), the higher-order modes should really just be governed by $du_{t}^{i,N}=-\alpha u_{t}^{i,N}dt$.

Hence, Propositions 9.1–9.2 and the multiscale nature of the problem induced by the trace-class noise suggests a procedure how to approximate the rate function and the associated LDP in practice. In particular, we may compute the eigenvalues and eigenfunctions of *K* and *Q* up to a sufficiently large given order $N^{*}$. This yields an explicit representation of the Galerkin system and the associated rate function. Then one may apply any finite-dimensional technique to understand the rate function. One may even find a better truncation order $N<N^{*}$ based on the knowledge that the minimizer of the rate function must have components that decay (almost) exponentially in time for orders bigger than *N*.

## 10 Outlook

In this paper, we have discussed several steps toward a better understanding of noise-induced transitions in continuum neural fields. Although we have provided the main basic elements via the LDP and finite-dimensional approximations, there are still several very interesting open problems.

We have demonstrated that a sharp Kramers’ rate calculation for neural fields with trace-class noise is very challenging as the techniques for white-noise gradient-structure SPDEs cannot be applied directly. However, we have seen in Sect. 4 that the deterministic dynamics for neural fields frequently exhibits a classical bistable structure with a saddle-state between stable equilibria. This suggests that there should be a Kramers’ law with exponential scaling in the noise intensity as well as a precisely computable pre-factor. It is interesting to ask how this pre-factor depends on the eigenvalues of the trace-class operator *Q* defining the *Q*-Wiener process. We expect that new technical tools are needed to answer this question.

From the viewpoint of experimental data, the exponential scaling for the LDP is relevant as it shows that noise-induced transitions have exponential interarrival times. This leads to the possibility that working memory as well as perceptual bistability could be governed by a Poisson process. However, the same phenomena could also be governed by a slowly varying variable, i.e., by an adaptive neural field [14]; the “fast” activity variable *U* in the Amari model is augmented by one or more “slow” variables. In this context, the required assumptions on the equilibrium structure in Sect. 4 and the noise in Sect. 3 is not necessary to produce a bistable switch and the fast variable *U* can, e.g., just have a single deterministically unstable equilibrium and bistable, nonrandom switching between metastable states may occur. Of course, there is also the possibility that an intermediate regime between noise-induced and deterministic escape is relevant [53]. 

It is interesting to note that the same problem arises generically across many natural sciences in the study of critical transitions (or “tipping points”) [48,59]. The question which escape mechanism from a metastable state matches the data is often discussed very controversially and we shall not aim to provide a discussion here. However, our main goal to make the LDP and its associated rate functional as explicit as possible should definitely help to simplify comparison between models and experiment. For example, a parameter study or data assimilation for the finite-dimensional Galerkin system considered in Theorem 8.1 and the associated rate function in Proposition 9.1 are often easier than working directly with the abstract solutions of the stochastic Amari model in $C([0,T],L^{2}(B))$.

To study the parameter dependence is an interesting open question, which we aim to address in future work. In particular, the next step is to use the Galerkin approximations in Sect. 8 and the associated LDP in Sect. 9 for numerical purposes [49]. Recent work for SPDEs [8] suggests that a spectral method can also be efficient for stochastic neural fields. Results on numerical continuation and jump heights for SDEs [47] can also be immediately transferred to the spectral approximation, which would allow for studies of bifurcations and associated noise-induced phenomena. 

One may also ask how far the technical assumptions we make in this paper can be weakened. It is not clear which parts of the global Lipschitz assumptions may be replaced by local assumptions or removed altogether. Similar remarks apply to the multiscale nature of the problem induced by the decay of the eigenvalues of *Q*. How far this observation can be exploited to derive more efficient analytical as well as numerical techniques remains to be investigated.

On a more abstract level, it would certainly be desirable to extend our basic framework to other topics that have been considered already for deterministic neural fields. A generalization to activity based models with nonlinearity $f(\int_{B}w(x,y)u(y)dy)$ seems possible. Furthermore, it may be highly desirable to go beyond stationary solutions and investigate noise-induced switching and transitions for traveling waves and patterns.

## Appendix: Convergence of the Galerkin Approximation

*Proof of Theorem 8.1* We fix a T>0. Throughout the proof an unspecified norm ∥⋅∥ or operator norm ⦀⋅⦀, respectively, are either for the Hilbert space $L^{2}(B)$ or the Banach space C(B) and estimates using the unspecified notation are valid in both cases. Furthermore, C>0 denotes an arbitrary deterministic constant, which may change from line to line but depend only on *T*. We begin the proof obtaining an a priori growth bound on the solution of the Amari equation (4). Using the linear growth condition on *F* implied by its Lipschitz continuity, we obtain the estimate

$$∥U_{t}∥\leq e^{-\alpha t}∥U_{0}∥+C\int_{0}^{t}e^{-\alpha (t-s)}\left(1+∥U_{s}∥\right)ds+∥O_{t}∥.$$

Due to Gronwall’s inequality, there exists a deterministic constant *C* such that it holds almost surely

(61) $$\underset{t\in [0,T]}{\sup}∥U_{t}∥\leq C\left(1+∥U_{0}∥+\underset{t\in [0,T]}{\sup}∥O_{t}∥\right)e^{CT}\;\text{a.s.}$$

Note that *O* is an Ornstein–Uhlenbeck process, and it thus holds

$$\underset{t\in [0,T]}{\sup}{∥O_{t}∥}_{L^{2}}<\infty$$

almost surely and under the assumptions of Lemma 2.1 in addition

$$\underset{t\in [0,T]}{\sup}{∥O_{t}∥}_{0}<\infty$$

almost surely.

Let $P^{N}$ denote the projection operator from $L^{2}(B)$ to the subspace spanned by the first *N* basis functions. Then we find that in Hilbert space notation the *N*th Galerkin approximation satisfies

$$U_{t}^{N}=e^{-\alpha t}P^{N}U_{0}+\int_{0}^{t}e^{-\alpha (t-s)}P^{N}KF\left(U_{t}^{N}\right)ds+\epsilon O_{t}^{N}.$$

Here, we use $O^{N}$ to be shorthand for the truncated stochastic convolution

(62) $$O_{t}^{N}:=\sum_{i=1}^{N}\lambda_{i}\int_{0}^{t}e^{-\alpha (t-s)}d\beta_{s}^{i}v_{i}.$$

Hence, we obtain for the error of the Galerkin approximation

$$\begin{array}{rcl} U_{t}-U_{t}^{N} & = & e^{-\alpha t}\left(U_{0}-P^{N}U_{0}\right)+\int_{0}^{t}e^{-\alpha (t-s)}\left(KF(U_{t})-P^{N}KF\left(U_{t}^{N}\right)\right)ds\\ & & +\epsilon \left(O_{t}-O_{t}^{N}\right). \end{array}$$

Adding and subtracting the obvious terms yields for the norm the estimate

$$\begin{array}{rcl} ∥U_{t}-U_{t}^{N}∥ & \leq & e^{-\alpha t}∥U_{0}-P^{N}U_{0}∥+⦀P^{N}K⦀\int_{0}^{t}e^{-\alpha (t-s)}∥F(U_{s})-F\left(U_{s}^{N}\right)∥ds\\ & & +⦀K-P^{N}K⦀\int_{0}^{t}e^{-\alpha (t-s)}∥F(U_{s})∥ds+\epsilon ∥O_{t}-O_{t}^{N}∥, \end{array}$$

where $⦀P^{N}K{⦀}_{L^{2}}\leq ⦀K{⦀}_{L^{2}}$ and $\sup_{N\in N}⦀P^{N}K{⦀}_{0}<\infty$ as a consequence of [[3], Lemma 11.1.4] (cf. the application of this result below). Next, using the Lipschitz and linear growth conditions on *F*, applying Gronwall’s inequality, taking the supremum over all t∈[0,T] and estimating using the bound (61) yield

(63) $$\begin{array}{rcl} & & \underset{t\in [0,T]}{\sup}∥U_{t}-U_{t}^{N}∥\\ & & \;\leq C\left(∥U_{0}-P^{N}U_{0}∥+⦀K-P^{N}K⦀\left(1+∥U_{0}∥+\underset{t\in [0,T]}{\sup}∥O_{t}∥\right)\right)\\ & & \;+C\left(\underset{t\in [0,T]}{\sup}∥O_{t}-O_{t}^{N}∥\right). \end{array}$$

It remains to show that the individual terms in the right-hand side converge to zero for N→∞ almost surely. 

– It clearly holds that ${∥U_{0}-P^{N}U_{0}∥}_{L^{2}}\to 0$ and the convergence ${∥U_{0}-P^{N}U_{0}∥}_{0}\to 0$ holds by assumption.

– Next, as argued above $\left(1+∥U_{0}∥+\sup_{t\in [0,T]}∥O_{t}∥\right)$ is a.s. finite and the compactness of the operator *K* implies $⦀K-P^{N}K⦀\to 0$ for N→∞, see [[3], Lemma 12.1.4]. 

– Finally, the third error term $\sup_{t\in [0,T]}∥O_{t}-O_{t}^{N}∥$ vanishes if the Galerkin approximations $O^{N}$ of the Ornstein–Uhlenbeck process *O* converge almost surely in the spaces $C([0,T],L^{2}(B))$ and C([0,T],C(B)), respectively. This convergence is proven in Lemma A.1 below.

 The proof is completed. □

The following lemma contains the convergence of the Galerkin approximation of the Ornstein–Uhlenbeck process necessary for proving Theorem 8.1.

**Lemma A.1***There exists a sequence*$b_{N}>0$*with*$\lim_{N\to \infty }b_{N}=0$*such that for all*T>0*and all*δ>0*there exists a random variable*$Z_{\delta }$*with*$E{\left|Z_{\delta }\right|}^{p}<\infty$*for all*p≥1*such that*

$$\underset{t\in [0,T]}{\sup}{∥O_{t}-O_{t}^{N}∥}_{L^{2}}\leq Z_{\delta }b_{N}^{1-\delta }$$

*almost surely*. *If*, *in addition*, *the series*$\sum_{i=1}^{\infty }\lambda_{i}^{2}v_{i}^{2}$*converges in*C(B)*and the functions*$v_{i}$*are Lipschitz continuous with Lipschitz constants*$L_{i}$*such that*$\sup_{x\in B}\sum_{i=1}^{\infty }\lambda_{i}^{2}L_{i}^{2\rho }{\left|v_{i}(x)\right|}^{2(1-\rho )}<\infty$*for a*ρ∈(0,1), *then it further holds that*

$$\underset{t\in [0,T]}{\sup}{∥O_{t}-O_{t}^{N}∥}_{0}\leq Z_{\delta }b_{N}^{1-\delta }$$

*almost surely*.

*Remark A.1* Assumptions on the speed of convergence of the series $\sum_{i=1}^{\infty }\lambda_{i}^{2}$ and $\sum_{i=1}^{\infty }\lambda_{i}^{2}v_{i}^{2}$ and $\sup_{x\in B}\sum_{i=1}^{\infty }\lambda_{i}^{2}L_{i}^{2\rho }{\left|v_{i}(x)\right|}^{2(1-\rho )}$ readily yield a rate of convergence for the Galerkin approximation due to the definition of the constants $b_{N}$ in the proof of the lemma.

*Proof of Lemma A.1* As in the proof Theorem 8.1 the unspecified norm ∥⋅∥ denotes either the norm in $L^{2}(B)$ or in C(B) and estimates are valid in both cases. We fix T>0, ρ∈(0,1) and a p∈N with p>2d/ρ. Throughout the proof C>0 denotes a constant that changes from line to line, but depends only on the fixed parameters *T*, *p*, *ρ*, *α* and the domain $B\subset R^{d}$.

Then we obtain for all N,M∈N with M<N using the factorization method (cf. [[23], Sect. 5.3]) similarly to the proof of [[8], Lemma 5.6] the estimate

$${\left(E\underset{t\in [0,T]}{\sup}{∥O_{t}^{N}-O_{t}^{M}∥}^{p}\right)}^{1/p}\leq C\underset{t\in [0,T]}{\sup}{\left(E{∥Y_{t}^{M,N}∥}^{p}\right)}^{1/p},$$

where $Y_{t}^{N,M}$ is the process defined by

$$Y_{t}^{M,N}=\sum_{i=M+1}^{N}\lambda_{i}\int_{0}^{t}{\left(t-s\right)}^{-\rho /2}e^{-\alpha (t-s)}d\beta_{s}^{i}v_{i}.$$

In order to estimate the *p*th mean of the process $Y^{M,N}$, we proceed separately for the two cases $L^{2}(B)$ and C(B).

*The case of*$L^{2}(B)$: Due to the orthogonality of the basis functions and employing Hölder’s inequality, one obtains

$$\begin{array}{rcl} & & E{∥Y_{t}^{M,N}∥}_{L^{2}}^{p}\\ & & \;=E{\left(\sum_{i=M+1}^{N}\lambda_{i}^{2}{\left(\int_{0}^{t}{\left(t-s\right)}^{-\rho /2}e^{-\alpha (t-s)}d\beta_{s}^{i}\right)}^{2}\right)}^{p/2}\\ & & \;=E{\left(\sum_{i=M+1}^{N}\lambda_{i}^{2(p-2)/p}{\left(\lambda_{i}^{2/p}\int_{0}^{t}{\left(t-s\right)}^{-\rho /2}e^{-\alpha (t-s)}d\beta_{s}^{i}\right)}^{2}\right)}^{p/2}\\ & & \;\leq E{\left({\left(\sum_{i=M+1}^{N}\lambda_{i}^{2}\right)}^{(p-2)/p}{\left(\sum_{i=M+1}^{N}{\left(\lambda_{i}^{2/p}\int_{0}^{t}{\left(t-s\right)}^{-\rho /2}e^{-\alpha (t-s)}d\beta_{s}^{i}\right)}^{p}\right)}^{2/p}\right)}^{p/2}\\ & & \;\leq {\left(\sum_{i=M+1}^{N}\lambda_{i}^{2}\right)}^{(p-2)/2}\sum_{i=M+1}^{N}E{\left(\lambda_{i}^{2/p}\int_{0}^{t}{\left(t-s\right)}^{-\rho /2}e^{-\alpha (t-s)}d\beta_{s}^{i}\right)}^{p}. \end{array}$$

Next, as the stochastic integrals in the right-hand side are centered Gaussian random variables [[8], Lemma 5.2]^c^ yields for all t≤T

$$\begin{array}{rcl} E{∥Y_{t}^{M,N}∥}_{L^{2}}^{p} & \leq & C{\left(\sum_{i=M+1}^{N}\lambda_{i}^{2}\right)}^{(p-2)/p}\sum_{i=M+1}^{N}\lambda_{i}^{2}{\left(\int_{0}^{t}{\left(t-s\right)}^{-\rho }e^{-2\alpha (t-s)}ds\right)}^{p/2}\\ & \leq & C{\left(\sum_{i=M+1}^{N}\lambda_{i}^{2}\right)}^{(p-2)/p}\sum_{i=M+1}^{N}\lambda_{i}^{2}{\left(\int_{0}^{T}s^{-\rho }e^{-2\alpha s}ds\right)}^{p/2}\\ & \leq & C{\left(\sum_{i=M+1}^{N}\lambda_{i}^{2}\right)}^{p/2}. \end{array}$$

Therefore, we obtain for all M,N∈N with M<N

(64) $${\left(\underset{t\in [0,T]}{\sup}E{∥Y_{t}^{N,M}∥}_{L^{2}}^{p}\right)}^{1/p}\leq C{\left(\sum_{i=M+1}^{N}\lambda_{i}^{2}\right)}^{1/2}\leq C{\left(\sum_{i=M+1}^{\infty }\lambda_{i}^{2}\right)}^{1/2},$$

where the final upper bound decreases to zero for M→∞ by assumption.

*The case of*C(B): In this case the estimates get a bit more involved. As ρ/2>d/p The continuous embedding of the Sobolev–Slobodeckij space $W^{\rho /2,p}(B)$ into C(B) (cf. [[58], Sect. 2.2.4 and 2.4.4]) and [[8], Lemma 5.2] yield the estimates

(65) $$\begin{array}{rcl} \underset{t\in [0,T]}{\sup}E{∥Y_{t}^{N,M}∥}_{0}^{p} & \leq & C\underset{t\in [0,T]}{\sup}\int_{B}\int_{B}\frac{E{\left|Y_{t}^{M,N}(x)-Y_{t}^{M,N}(y)\right|}^{p}}{{\left|x-y\right|}^{d+\rho p/2}}dxdy\\ & & +C\underset{t\in [0,T]}{\sup}\int_{B}E{\left|Y^{M,N}(x)\right|}^{p}dx\\ & \leq & C\underset{t\in [0,T]}{\sup}\int_{B}\int_{B}\frac{{\left(E{\left|Y_{t}^{M,N}(x)-Y_{t}^{M,N}(y)\right|}^{2}\right)}^{p/2}}{{\left|x-y\right|}^{d+\rho p/2}}dxdy\\ & & +C\underset{t\in [0,T]}{\sup}\int_{B}{\left(E{\left|Y^{M,N}(x)\right|}^{2}\right)}^{p/2}dx. \end{array}$$

We proceed estimating the two expectation terms in the right-hand side. Then we obtain for all M<N and all x,y∈B for the first term

(66) $$\begin{array}{rcl} & & E{\left|Y_{t}^{M,N}(x)-Y_{t}^{M,N}(y)\right|}^{2}\\ & & \;=E{\left|\sum_{i=M+1}^{N}\lambda_{i}\int_{0}^{t}{\left(t-s\right)}^{-\rho /2}e^{-\alpha (t-s)}d\beta_{s}^{i}\left(v_{i}(x)-v_{i}(y)\right)\right|}^{2}\\ & & \;\leq \sum_{i=M+1}^{N}\lambda_{i}^{2}\int_{0}^{T}s^{-\rho }e^{-2\alpha s}ds{\left|v_{i}(x)-v_{i}(y)\right|}^{2}\\ & & \;\leq C\sum_{i=M+1}^{N}\lambda_{i}^{2}L_{i}^{2\rho }{\left|x-y\right|}^{2\rho } \end{array}$$

for any ρ∈(0,1) and for the second term

(67) $$\begin{array}{rcl} E{\left|Y_{t}^{M,N}(x)\right|}^{2} & \leq & \sum_{i=M+1}^{N}\lambda_{i}^{2}\int_{0}^{t}{\left(t-s\right)}^{-\rho }e^{-2\alpha (t-s)}dsv_{i}{\left(x\right)}^{2}\\ & \leq & C\sum_{i=M+1}^{N}\lambda_{i}^{2}v_{i}{\left(x\right)}^{2}. \end{array}$$

Next applying the estimates (67) and (66) to the right-hand side of (65) yields, note that ρp/2−d>0,

$$\begin{array}{rcl} {\left(\underset{t\in [0,T]}{\sup}E{∥Y_{t}^{N,M}∥}_{0}^{p}\right)}^{1/p} & \leq & C(\int_{B}\int_{B}\frac{{\left(\sum_{i=M+1}^{N}\lambda_{i}^{2}L_{i}^{2\rho }{\left|x-y\right|}^{2\rho }\right)}^{p/2}}{{\left|x-y\right|}^{d+\rho p/2}}dxdy\\ & & +{\int_{B}{\left(\sum_{i=M+1}^{N}\lambda_{i}^{2}v_{i}{\left(x\right)}^{2}\right)}^{p/2}dx)}^{1/p}\\ & \leq & C{(\int_{B}\int_{B}{\left|x-y\right|}^{\rho p/2-d}dxdy\left(\sum_{i=M+1}^{N}\lambda_{i}^{2}L_{i}^{2\rho }\right)}^{p/2}\\ & & +{{\left(\underset{x\in B}{\sup}\left|\sum_{i=M+1}^{N}\lambda_{i}^{2}v_{i}{\left(x\right)}^{2}\right|\right)}^{p/2})}^{1/p}\\ & \leq & C{\left(\underset{x\in B}{\sup}\left|\sum_{i=M+1}^{N}\lambda_{i}^{2}v_{i}{\left(x\right)}^{2}\right|+\sum_{i=M+1}^{N}\lambda_{i}^{2}L_{i}^{2\rho }\right)}^{1/2} \end{array}$$

for any ρ∈(0,1). Due to the assumptions of the lemma the two summations in the right hand side converge for N→∞, and thus we obtain for all M,N∈N with M<N the estimate

(68) $${\left(\underset{t\in [0,T]}{\sup}E{∥Y_{t}^{N,M}∥}_{0}^{p}\right)}^{1/p}\leq C{\left(\underset{x\in B}{\sup}\left|\sum_{i=M+1}^{\infty }\lambda_{i}^{2}v_{i}{\left(x\right)}^{2}\right|+\sum_{i=M+1}^{\infty }\lambda_{i}^{2}L_{i}^{2\rho }\right)}^{1/2},$$

where the right-hand side decreases to zero for M→∞.

Overall, we infer from the estimates (64) and (68) that $O^{N}$ is a Cauchy-sequence in the two spaces $C([0,T],L^{2}(B))$ and C([0,T],C(B)) with respect to convergence in the *p*th mean and the limit is given by the process *O*. Moreover, it holds that

(69) $${\left(E\underset{t\in [0,T]}{\sup}{∥O_{t}-O_{t}^{N}∥}^{p}\right)}^{1/p}\leq Cb_{N}\;\forall N\in N,$$

where the constant *C* depends only on *p* but is independent of *N* and the sequence $b_{N}$ is independent of *p* and $\lim_{N\to \infty }b_{N}=0$. As we fixed p∈N sufficiently large at the beginning of the proof, the result (69) holds for all sufficiently large p∈N. Then, however, Jensen’s inequality implies that (69) holds for all p∈[1,∞). Proceeding as in the proof of [[44], Lemma 2.1] using the Chebyshev–Markov inequality and the Borel–Cantelli lemma, one obtains that there exists for all δ>0 a random variable $Z_{\delta }$ with $E{\left|Z_{\delta }\right|}^{p}<\infty$ for all p≥1 such that

(70) $$\underset{t\in [0,T]}{\sup}∥O_{t}-O_{t}^{N}∥\leq Z_{\delta }b_{N}^{1-\delta }\;\text{almost surely}.$$

The proof is completed. □

## Competing Interests

The authors declare that they have no competing interests.

## Authors’ Contributions

Both authors contributed equally to the paper.
